# Tracheal agenesis as a rare cause of difficult intubation in a newborn with respiratory distress: a case report

**DOI:** 10.1186/1752-1947-3-105

**Published:** 2009-11-04

**Authors:** Raja Ahmad, Kahairi Abdullah, Lukman Mokhtar, Ahmad Fadzil

**Affiliations:** 1 Department of Otolaryngology - Head & Neck Surgery, Faculty of Medicine, International Islamic University Malaysia, Kuantan 25200, Malaysia; 2 Department of Anaesthesiology and Intensive Care, Faculty of Medicine, International Islamic University Malaysia, Kuantan 25200, Malaysia; 3 Department of Pediatric, Tengku Ampuan Afzan Hospital, Kuantan 25710, Malaysia

## Abstract

**Introduction:**

Tracheal agenesis is a very rare congenital airway anomaly. It may pose a great challenge to the first attending physician both in diagnosis and in establishing the airway during the first day of life.

**Case presentation:**

We report a newborn Malay baby boy with trachea agenesis (type III by Floyd's classification) who presented with severe respiratory distress immediately after birth. Clinical diagnosis in this case was not straightforward, as it started with difficulty in intubation followed by an unsuccessful emergency tracheostomy in the neonatal intensive care unit. Urgent surgical neck exploration with endoscopic examination in the general operating theatre revealed the final diagnosis. The authors present a short description of the embryopathology and diagnostic criteria of the abnormality.

**Conclusion:**

We hope this case presentation will be valuable in increasing the awareness of physicians about this rare cause of tracheal obstruction or difficult intubation.

## Introduction

Tracheal agenesis is a rare congenital airway anomaly. There were 116 cases of tracheal agenesis reported in the literature between 1900 and (September) 2004 [1]. Airway management in this abnormality poses a great challenge to otolaryngologists, anaesthetists and pediatricians. This condition is incompatible with life. At present there is no specific surgical management technique that is associated with survival of tracheal agenesis.

The newborn with tracheal agenesis usually presents with immediate and severe respiratory distress, and the only way to provide ventilation is through the esophagus. Performing endotracheal intubation or tracheostomy does not normally help. Bag-mask ventilation and intubation of the esophagus may allow ventilation of the lungs. Neck exploration during the tracheostomy and endoscopic evaluation will establish the diagnosis. The ex-utero intrapartum treatment (EXIT) procedure is an excellent method developed for use in anticipation of possible airway compromises in newborn babies at birth.

## Case presentation

A 2.4 kg Malay baby boy was delivered after 37 weeks of gestation by spontaneous vaginal delivery. The pregnancy was complicated by polyhydramnios. The baby developed immediate respiratory distress at birth with an Apgar score of 1 at 1 min, 5 at 5 min, and 6 at 10 minutes of life. He was initially resuscitated with bag-mask ventilation, and subsequently transferred to the neonatal intensive care unit (NICU). Multiple oral endotracheal intubations were attempted in the NICU with no success. Bag-mask ventilation was continued and an otolaryngologist was consulted for emergency tracheostomy. Oxygen saturation was successfully maintained at above 85% with bag-mask ventilation. It was possible to pass a Ryle's tube through both nostrils.

**Figure 1:**
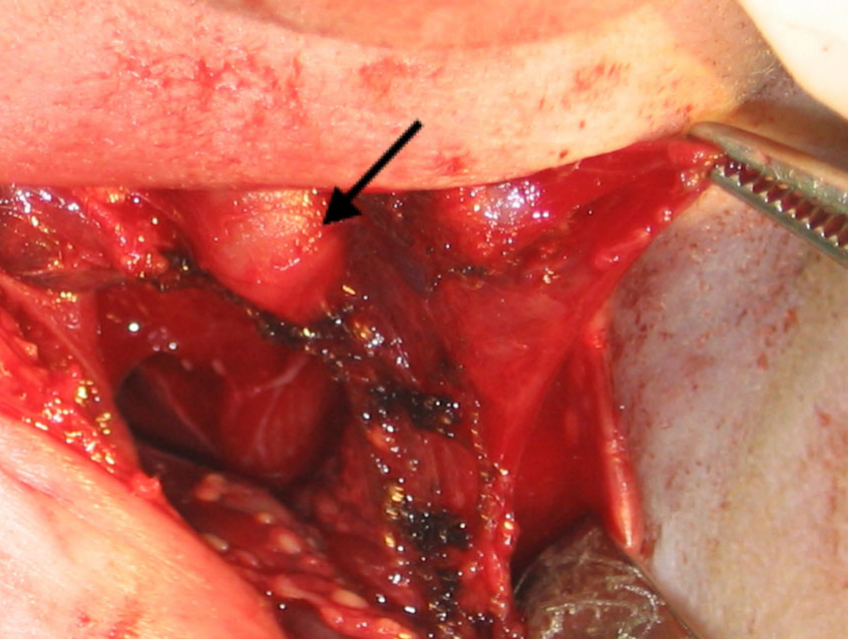
**Neck exploration revealed a normal larynx that ended in a blind pouch at the level of cricoid (black arrow)**. The dome-shaped cricoid occludes the lumen.

**Figure 2:**
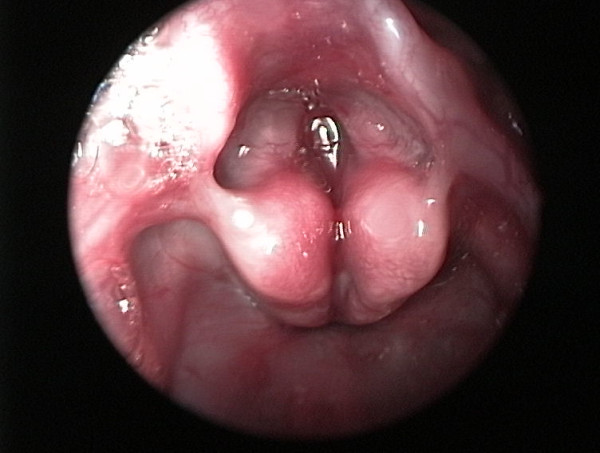
**Laryngoscopy examination revealed a cleft larynx with immobile vocal cords bilaterally in midline position**.

**Figure 3:**
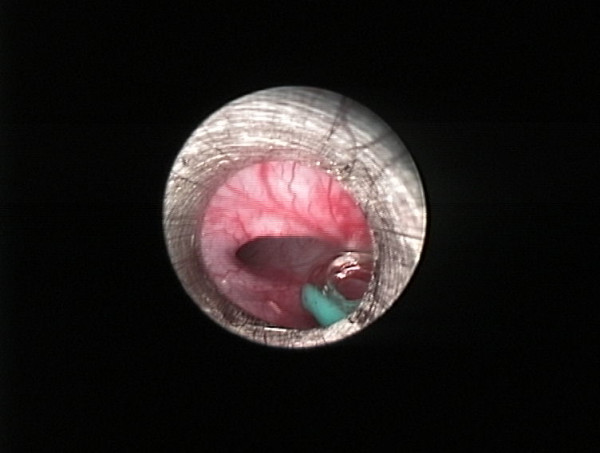
**Esophagoscopy view showing one of the openings into the left bronchus at the distal end of the esophagus**.

During the surgical procedure, the trachea could not be identified. The baby was transferred to the operating theatre for neck exploration, and a complete endoscopic examination was performed to evaluate airway patency. During the neck exploration, it was noted that the larynx ended blindly at the cricoid level (Figure 1), while the trachea was absent. A laryngoscopic evaluation without muscle relaxant disclosed a cleft larynx with bilateral immobile vocal cords (Figure 2). A bronchoscope could not be passed below the vocal cords. An esophagoscopy was performed which revealed two openings at the distal portion of the esophagus, which communicated with the left and right bronchus, respectively (Figure 3).

At the end of the surgical procedure, an endotracheal tube was inserted into the esophagus and effective ventilation confirmed by visualization of normal chest expansion and good oxygen saturation. The baby also had persistent ductus arteriosus, dysplasia of the right radius and the right thumb, and an imperforated anus. A diagnosis of tracheal agenesis was made and the family members were counseled about the grim prognosis. Life support was discontinued with the agreement of the parents, and the baby was allowed to die.

## Discussion

At present, there is no specific surgical management that allows survival in cases of tracheal agenesis. Normally, a newborn with tracheal agenesis presents with immediate respiratory distress and an absent or very weak cry. This rare congenital anomaly might confound the attending doctor in the delivery room or operating theatre.

The embryopathology resulting in this abnormality occurs during the first eight weeks of gestation. The tracheo-pulmonary complex develops from the respiratory diverticulum at the ventral aspect of the primitive foregut. A compromised vascular supply to the developing trachea during this stage may cause tracheal agenesis or tracheal stenosis with complete tracheal ring [2]. According to Merei *et al *[3], the point of bifurcation between the developing trachea at ventral and developing esophagus at dorsal foregut remains fixed in relation to the cervical vertebra. Caudally, the respiratory diverticulum will develop into the carina and broncho-pulmonary tree. The cephalic aspect of the respiratory diverticulum will be elongated to form the trachea and the infra-glottic structure. Tracheal agenesis results when this normal elongation process fails to take place [1]. This anomaly is associated with relatively normal supra-glottic structures and pulmonary development, as seen in this case. The congenital abnormality is only limited to the region of the developing trachea. The severity of tracheal agenesis was described in detail by Floyd and colleagues and classified into three types [4]. In Type I, a short segment of the trachea fails to elongate to fuse with the larynx. In Type II, the respiratory diverticulum fuses in midline to form the carina but tracheal elongation does not take place. And in type III, the respiratory diverticula does not fuse in the midline, resulting in two fistula opening at the lower part of esophagus. The present case is compatible with a type III abnormality. Type II remains the most common abnormality (61%), followed by type III (23%) and type I (11%) [1].

Tracheal agenesis is commonly associated with other congenital anomalies such as vertebral defects, anal atresia, tracheoesophageal fistula, esophageal atresia, cardiovascular defects, limb defects, duodenal atresia and renal defects. Tracheal agenesis can be a manifestation of several syndromes such as VATER (vertebrae, anus, trachea, esophagus, and renal), also known as VACTERL, and TARCD (total alkaloids from rhizoma corydalis decumbeutis).

A high index of suspicion is required to diagnose tracheal atresia. Antepartum features that would corroborate such suspicion are the presence of polyhydramnios with multiple fetal anomalies. During birth, the baby may not cry or may have a weak cry. An acute severe respiratory distress develops and multiple attempts at intubations fail. Laryngoscopy will reveal immobile vocal cords lying in the midline position. Other findings are a cleft between the arytenoids, as well as associated congenital anomalies. Good oxygenation may be maintained with bag-mask ventilation or esophageal intubation. The diagnosis is made through neck exploration during emergency tracheostomy and an endoscopic evaluation of the larynx and esophagus. A pre-delivery procedure with three-dimensional ultrasound or fetal magnetic resonance imaging allows a complete evaluation of this upper airway abnormality. The ex-utero intrapartum treatment (EXIT) procedure can be planned based on the imaging results. EXIT procedure can reduce the risk of respiratory distress immediately after birth [5].

## Conclusion

Tracheal agenesis should be suspected in a newborn baby who presents with immediate respiratory distress, as well as extremely weak cry and failed intubation despite adequate ventilation with facemask. The establishment of airway after an insertion of endotracheal tube in the oesophagus will further enhance the index of suspicion before the definitive endoscopic evaluation.

## Consent

Written informed consent could not be obtained in this case since the patient is now lost to follow-up. We believe that this case report contains a worthwhile clinical lesson which could not be made as effectively in any other way. We expect that the patient and her family would not object to the publication since every effort has been made so that she remains anonymous.

## Competing interests

The authors declare that they have no competing interests.

## Authors' contributions

All authors of this paper have participated directly in the planning, execution, or analysis of this report, and have read and approved the final version submitted.
